# Review of Health Hazards in High-Entropy Alloy Processing Under Laboratory Conditions and Risk Assessment Using a Simple Risk Scoring Model

**DOI:** 10.3390/toxics13090777

**Published:** 2025-09-13

**Authors:** Sheetal Kumar Dewangan

**Affiliations:** Department of Materials Science and Engineering, Ajou University, Suwon 16499, Republic of Korea; sheetal@ajou.ac.kr

**Keywords:** health hazard, metal powder processing, powder metallurgy, high-entropy alloy, risk scoring model

## Abstract

Certain metal powders used in the synthesis and processing of high-entropy alloys (HEAs) pose significant health hazards, as many of these metals are toxic substances with no biological role in the human body. These metals can mimic essential elements or interfere with metabolic processes (the chemical reactions in living organisms that sustain life), leading to detrimental health effects. While some metals, such as aluminum, can be eliminated from the body through natural biological processes, others tend to accumulate, causing chronic illnesses over time. This review examines the toxicity mechanisms and health impacts of metals used in HEA synthesis, focusing on laboratory-scale processing. It also identifies potential health risks associated with occupational exposure in laboratory environments, including the inhalation of toxic metal powders and nanoparticles. A simple risk scoring model is introduced to systematically assess and quantify these risks based on factors such as toxicity levels, exposure limits, and carcinogenic potential (the ability of a substance to cause cancer) as given by the Occupational Safety and Health Administration (OSHA) and the National Institute for Occupational Safety and Health (NIOSH) standards. The proposed model can be applied to evaluate the relative hazards of commonly used HEA constituent powders (e.g., Ni, Co, Cr, and Al), offering practical guidance for safer laboratory handling and material selection. By integrating this risk assessment framework, this review aims to enhance workplace safety, guide the development of better material handling practices, and assist researchers in mitigating health risks associated with HEA processing.

## 1. Introduction

In today’s technologically advanced world, most metals find widespread use as alloys. However, some metals are used in their pure elemental form for engineering purposes. An alloy is defined as a macroscopically homogeneous metallic composition comprising two or more elements mixed in a manner that makes them difficult to separate easily by mechanical means. This definition is accepted worldwide (UN GHS, 2015) [1,2]. Thus, alloys must be considered as “special mixtures” rather than as simple mixtures, where the addition of constituent elements causes the alloy’s chemical matrix to change its properties. The physical, mechanical, and chemical properties of different alloys are distinct and vary considerably from those of the constituent metals.

As a result, assessing the health-related and environmental hazards of an alloy based on its constituent elements may produce inaccurate results. Instead, alloys should be evaluated according to their inherent composite properties. Specifically, the following factors must be considered when classifying or analyzing hazardous alloys: (i) Alloys display distinctive properties and intrinsic hazard profiles different from those of their constituent elements. (ii) Identification of the hazards and determination of the toxicity of an alloy depend strongly on the alloy’s rate of transformation/dissolution (TD) into its bioaccessible form [3,4].

As with metals and metal complexes, speciation is important for alloys. Components of released metals may exist in a range of valences, linked to different anions or cations. Variations in bioavailability (the proportion of a substance that enters circulation and is available for biological activity) might arise from released ions binding to adsorptive substances in the environment, such as mineral components in sediments or soils, or dissolved organic matter in water. As discussed in the following section, speciation and valence are significant factors in determining toxicity, as is the differentiation of released metal ions. Hence, considering the wide variety of alloys currently in existence, grouping alloys and utilizing read-across techniques within an alloy group are essential for effective hazard assessment, which is critical for ensuring human well-being and ecological preservation [5,6,7,8].

In brief, alloys possess unique properties and pose hazards that differ from those of their constituent elements. Therefore, evaluating alloys based on their intrinsic composite characteristics and considering factors such as TD rates and speciation is essential for accurate hazard assessment and risk management [9,10].

Certain metal powders used in manufacturing processes and other applications can pose significant health risks. Lead, for example, can be easily absorbed through the lungs and can act as a potent toxin within the body. Similarly, inhaling cadmium particles produced during metal spraying can lead to toxic effects. Furthermore, certain metals have been found to trigger allergic reactions. Cobalt, for instance, has been linked to acute asthma in hypersensitive individuals and has also been identified as the likely cause of slow, progressive lung fibrosis observed in lab personnel exposed to tungsten carbide [11,12,13,14].

Aluminum powders, commonly used in fireworks, have been known to cause severe lung fibrosis that develops rapidly. Additionally, when deposited in the lungs, metals such as tin and iron generally do not cause immediate harm but can create distinct shadows when viewed through chest radiographs. Preventing these adverse health effects requires awareness of the potential hazards associated with metal powders and strict adherence to permissible threshold limit values (TLVs) for such powders in the atmosphere. In certain situations, medical supervision is advisable for ensuring the well-being of individuals exposed to such hazards [15,16]. In addition, some metals commonly used in powder metallurgy are relatively harmless when their dust is inhaled. Iron, a natural component of hemoglobin in the blood, can be inhaled by welders, emery polishers, and hematite miners. Although iron deposits in the lungs can cause noticeable shadows in X-ray scans, no evident health hazards have been associated with these deposits, as they are gradually reabsorbed into the body. In contrast, inhaling zinc fumes can result in a condition known as metal fume fever, which produces malaria-like symptoms—including fever and a general sense of illness—that manifest for several hours after the fine particles in the fumes have been inhaled [17,18,19,20]. This condition was prevalent among brass casters in the past and can occur sporadically during the welding of zinc-coated metal or metal spraying.

Metallic nickel has the potential to cause allergic rashes, such as the commonly observed “suspender rash” resulting from prolonged contact with suspenders on the thigh. Lab personnel handling nickel-plated objects or inhaling nickel fumes during milling processes can develop a high degree of sensitivity to such rashes. In Canada and Norway, an increased incidence of nose and lung disease has been observed among individuals involved in the production of nickel using electrolytic processes. Although no conclusive evidence has been established, there is suggestive evidence of carcinogenicity, as metallic nickel dust is classified by IARC as Group 2B (possibly carcinogenic to humans) [21,22,23]. Moreover, chrome, like nickel, can cause allergic rashes as well as ulcers on the skin and nasal septum. Lung cancer has been associated with the manufacture of chromate compounds and is suspected of being linked with exposure to certain chrome pigments. While metallic chromium dust itself is not classified as carcinogenic to humans, hexavalent chromium compounds (Cr(VI)) are IARC Group 1 carcinogens and pose significant health risks [24].

Pneumoconiosis is a condition wherein the lung reacts to dust by creating fibrous tissue, and it can be induced by graphite dust. Graphite materials with relatively high silica levels can exacerbate this condition. In addition to burning the nose and throat and turning the skin a greenish-black hue, copper dust, in high doses, has been known to induce metal fume fever. The usage of aluminum powder in pyrotechnics has been reported to be associated with the development of acute lung fibrosis among workers in one manufacturing plant and a delayed onset of fibrosis among workers in another plant. These observations appear to be specific to the aforementioned locations and may be related to certain manufacturing and coating processes that utilize aluminum powder [15,16,25].

In the past, beryllium has been used in powder metallurgy in Germany. Even at very low concentrations, exposure to this metal and its salts can cause berylliosis, also known as beryllium illness, especially in vulnerable people. In severe cases of berylliosis, nodules are formed in the lungs and other organs, and noticeable weight loss occurs [26,27]. Cobalt is a metal that is commonly used as a binder, along with other metals such as vanadium, molybdenum, and even the aforementioned beryllium. Extensive research has demonstrated that workers involved in the manufacture of hard metals and metallic compounds, particularly tungsten carbide, tend to suffer from respiratory disorders, lung anomalies, and an elevated risk of lung cancer, with hard-metal dust containing cobalt and tungsten carbide classified by IARC as Group 2A (probably carcinogenic to humans). Specifically, coughing and tightness in the chest are common symptoms that may appear at any point after starting employment [28,29]. This hazard was discovered when a previously healthy individual, who underwent yearly chest X-ray observations at a clinic, displayed significant changes upon beginning work with hard metals [30,31]. This discovery prompted a search for similar cases among past attendees at the clinic. Subsequently, with the cooperation of the relevant companies and the Medical Research Council, approximately 250 workers from six factories underwent radiological examinations, with a subset of them also undergoing simple breathing tests. The investigation identified five cases of X-ray abnormalities among hard-metal manufacturers and one case among tool grinders. In one of these cases, where the individual had died of coincidental lung cancer, the lungs exhibited distinct fibrous changes in tissue [32,33,34,35,36].

Lead, a toxic metal, produces multiple toxic effects upon absorption into the body. It attaches to the surface of red blood cells and can accumulate in bones in a manner similar to calcium. It is excreted through the bowels and kidneys. Lead affects the synthesis of hemoglobin, leading to anemia and abnormalities in red blood cells. In industrial environments, inhalation is the primary route through which lead is absorbed into the body. While lead poisoning may cause abdominal pain and colic, these symptoms are relatively uncommon. Severe poisoning resulting in nerve damage and brain impairment is now rare but has been observed in the past among individuals exposed to lead spraying. Cadmium is another metal that can be absorbed into the body, transported through the bloodstream, stored in the liver, and ultimately excreted [5,37]. In addition, each element has specific occupational exposure limits and associated health effects that can be described in Table 1. Below is a summary of the permissible exposure limits (PEL) set by the Occupational Safety and Health Administration (OSHA) [38], recommended exposure limits (REL) from the National Institute for Occupational Safety and Health (NIOSH) [39], and primary health effects for several common metals found in HEA processes:

In general, alloys are composed of a base element, which is typically a metal or a metalloid, constituting the majority of the material, along with one or more added elements. These additions are made to achieve specific and improved mechanical, physical, or chemical properties compared with those of the individual components of the alloy. One of the earliest examples of alloy usage is the production of bronze by combining copper and tin, around 3000 BC. Steel, which is predominantly composed of iron and carbon, with varying amounts of other elements, is the most commonly used alloy. Almost all aluminum products are alloys of aluminum containing small amounts of other metals such as iron, silicon, magnesium, copper, zinc, and manganese, which are added depending on the desired properties for the intended use of the alloy [40,41]. In fact, the majority of metallic materials we encounter in our daily lives are alloys [42].

High-entropy alloys (HEAs) have gained significant attention due to their unique combination of properties that make them promising candidates for various industrial applications. Their exceptional mechanical strength, high thermal stability, corrosion resistance, and oxidation resistance enable their use in demanding environments. For instance, HEAs are being explored in aerospace and aviation industries for high-temperature structural components, in nuclear reactors for radiation-resistant materials, in the energy sector for advanced turbine and power generation systems, and in defense applications requiring lightweight yet durable alloys [43]. These emerging applications highlight the technological relevance of HEAs and provide strong motivation for systematically addressing the health and safety risks associated with their synthesis and processing, particularly at the laboratory scale where researchers are frequently exposed to hazardous metal powders [44].

Alloys are processed using various techniques, which involve modifying the structure and properties of the material. Alloys are often produced to achieve enhanced characteristics such as strength, durability, corrosion resistance, or electrical conductivity. The processing of alloys aims to optimize these properties according to the requirements of specific applications. Some common methods for processing alloys include melting, casting, and powder metallurgy [45]. The initial step in processing alloys is usually melting the constituent metals in a furnace and then pouring the molten alloy into molds to create castings. By contrast, powder metallurgy involves the fabrication of alloys from fine metal powders. Techniques such as compaction, sintering, and hot isostatic pressing are used to consolidate the powders into solid masses with controlled porosity and desired properties [46,47]. The selection of appropriate processing methods depends on factors such as alloy composition, intended application, production scale, and economic considerations [48].

While such processing considerations are well established for conventional alloys, high-entropy alloys (HEAs) introduce additional complexity. The novelty of this review lies in systematically linking these well-established occupational hazards with the unique compositional complexity, processing routes, and emerging toxicological concerns specific to HEAs. Unlike conventional alloys, HEAs involve simultaneous handling of multiple hazardous elements (such as Ni, Co, Cr, and refractory metals), where synergistic or cumulative toxic effects may arise.

## 2. Background of HEAs

**Figure 1:**
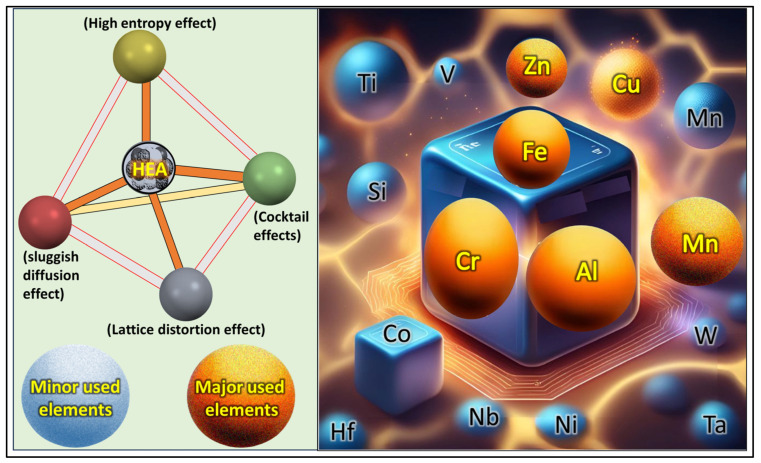
Schematic representation of major properties of HEAs, including fundamental properties and the most common hazardous elements used for the synthesis.

The novel concept of high-entropy alloys (HEAs) emerged in 2004, marking a new approach in alloy design [48]. These alloys consist of five or more principal elements with equiatomic or nearly equiatomic concentrations of 5–35% at. In addition, HEAs exhibit better strength, fracture toughness, thermal stability, corrosion resistance, and wear resistance compared with conventional alloys [49]. However, given the wide range of possible compositions, the major challenge with producing HEAs is identifying the ideal composition range to achieve the required properties. Thus, several experimental studies have attempted to discover novel HEAs and evaluate their properties [49,50,51]. The fundamental concept and major elements used in the processing of the HEAs are presented in Figure 1.

**Figure 2:**
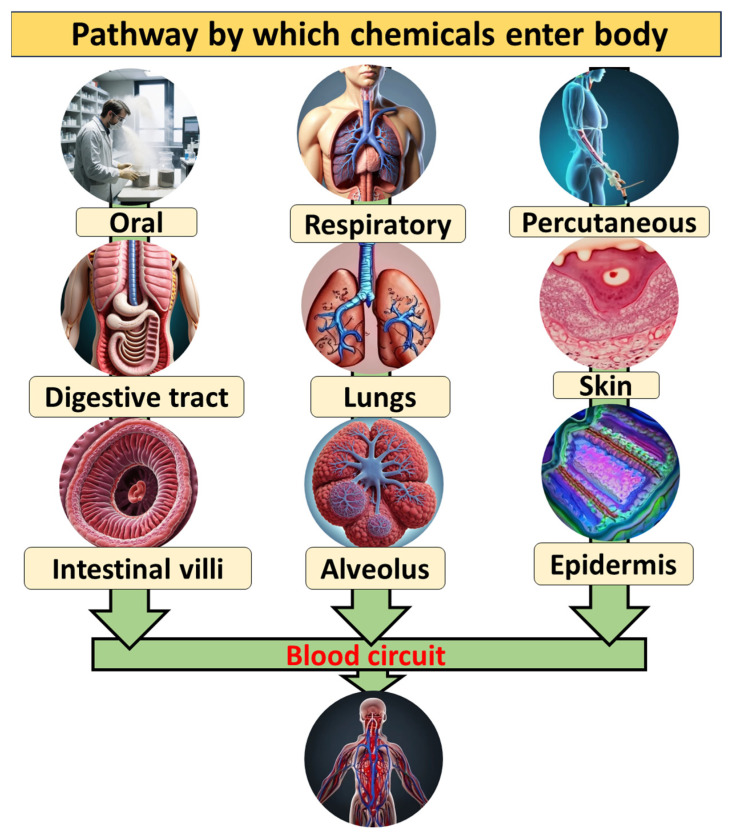
Schematic presentation of the Pathway by which chemicals enter body.

Chemicals used in powder metallurgy labs can enter the body through four main ways: ingestion, inhalation, skin contact, and eye contact. Fine metallic powders and fumes can become airborne during a variety of processes, entering the respiratory system and possibly causing irritation or long-term lung conditions, which makes inhalation a serious concern. Handling powders and chemicals directly or through contaminated surfaces can result in skin contact, which can cause skin absorption and potentially cause irritation or allergic reactions. The detailed pathway for entering the body is given in Figure 2.

**Figure 3:**
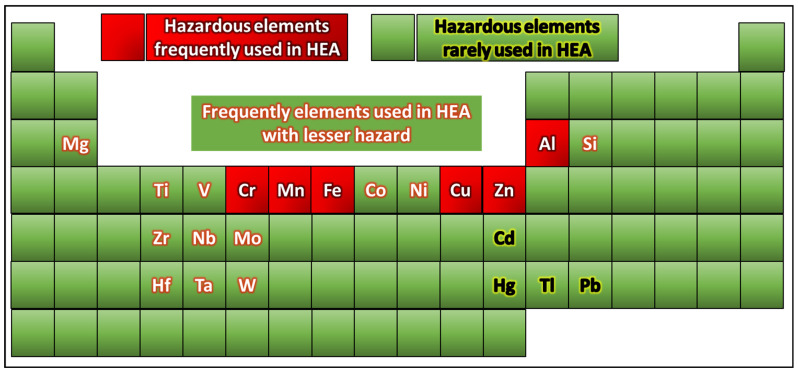
Periodic table of the most common hazardous elements used for the synthesis of HEAs.

Notably, metallurgy and materials science are interdisciplinary fields, where individuals from the constituent branches may not be aware of the hazards associated with the metals and materials used during the processing of alloys. To mitigate these hazards, it is important to implement appropriate safety measures, including adequate training for the personnel involved in alloy preparation, provisions such as local exhaust ventilation systems to control dust and fume exposure, and appropriate personal protective equipment (PPE) to protect against heat, chemicals, dust, or fumes. Moreover, proper storage, handling, and disposal procedures for hazardous materials and compliance with relevant safety regulations and standards are essential. It is also important to consult material safety data sheets (MSDS) or seek guidance from experts or safety professionals to ensure safe practices during alloy preparation [52]. A periodic table highlighting different hazardous elements is shown in Figure 3.

The objective of this review is to investigate the processing methods for HEAs within laboratory environments and assess potential health hazards associated with the fabrication, handling, and disposal of HEAs. This includes evaluating the exposure risks faced by laboratory personnel, understanding the toxicity mechanisms (the biological processes through which harmful effects of metals occur) and harmful effects of HEA materials, and proposing mitigation strategies to ensure occupational safety and self-protection.

### Classes of Elements Used in HEAs

**Figure 4:**
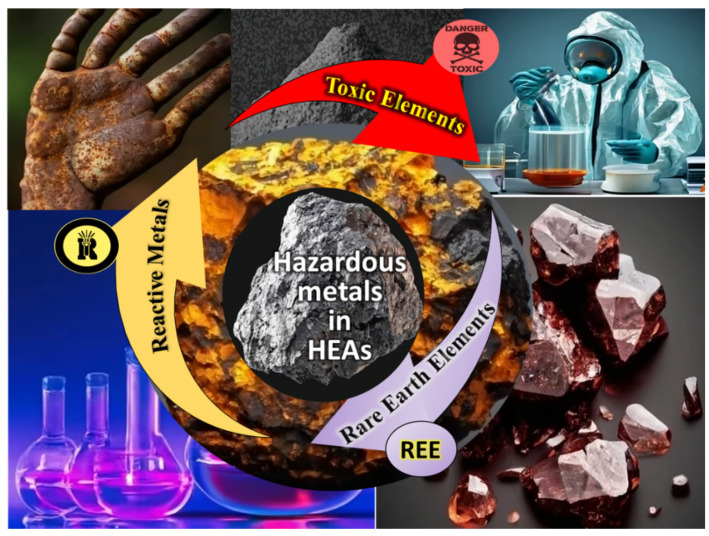
Different classes of hazardous metals are used to synthesize HEAs.

**Figure 5:**
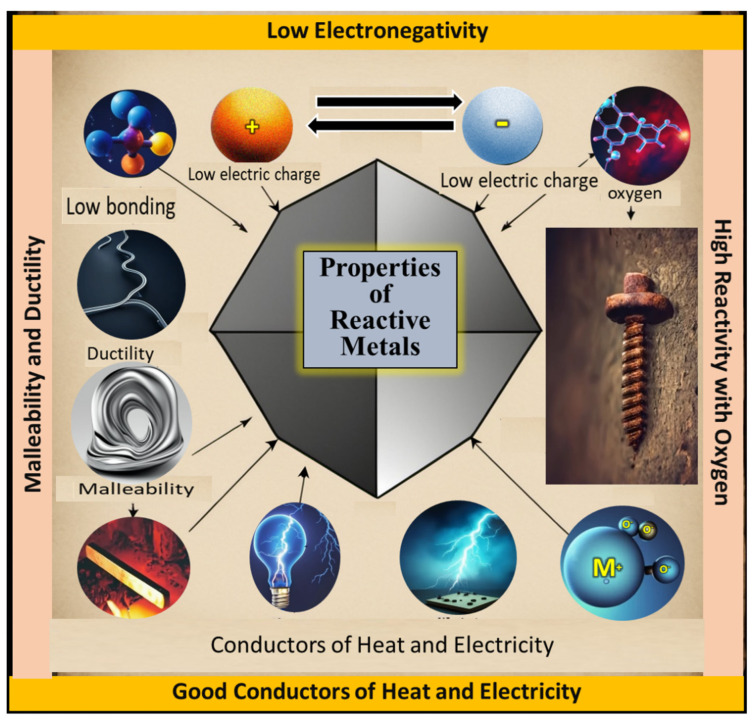
Major properties of reactive metals include low electronegativity, high reactivity with oxygen, malleability, ductility, low bonding strength, and good electrical and thermal conductivity.

**Figure 6:**
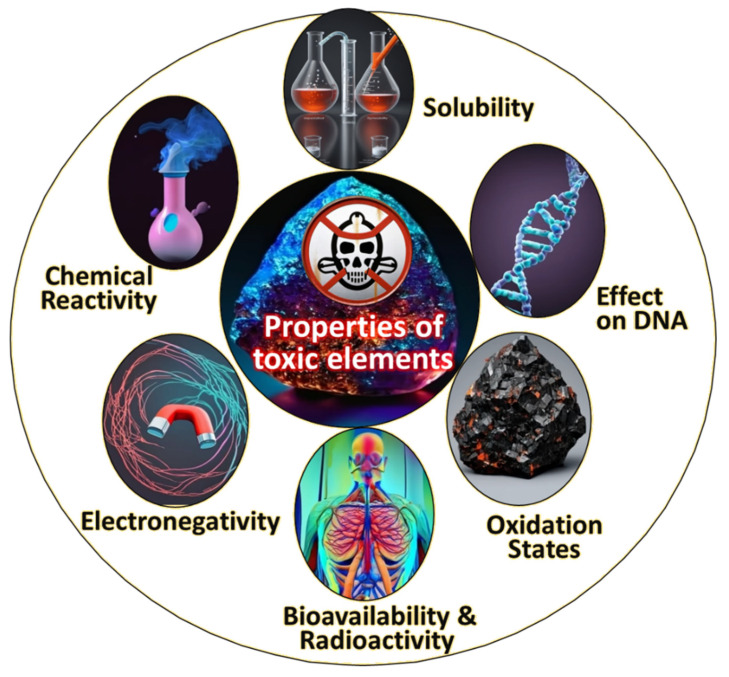
Major properties of toxic elements, including chemical reactivity, solubility, electronegativity, oxidation states, bioavailability, radioactivity, and their adverse effects on DNA.

**Figure 7:**
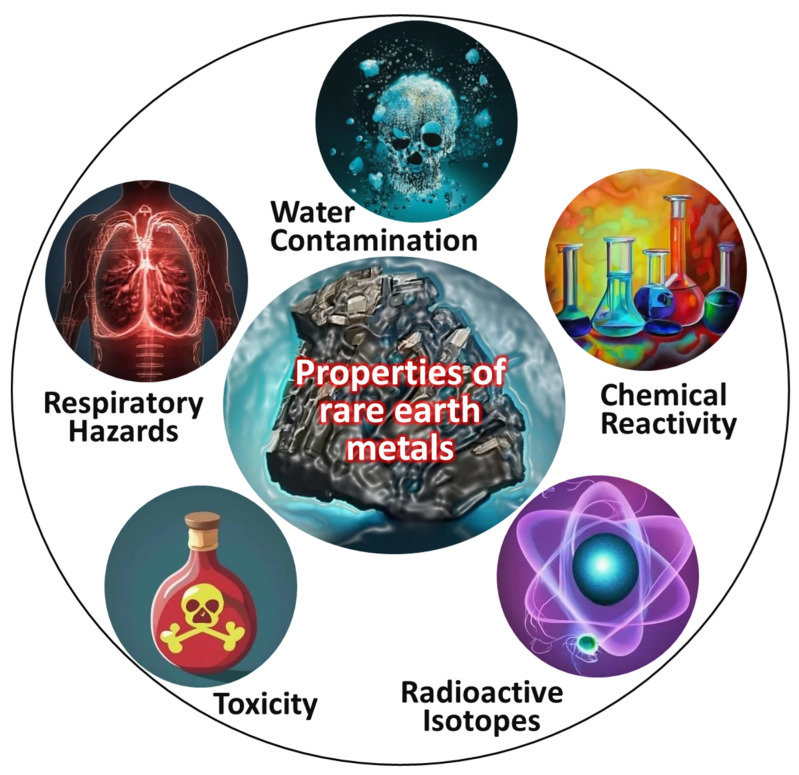
Major properties of rare-earth metals that pose health and environmental hazards, including water contamination, chemical reactivity, radioactive isotopes, toxicity, and respiratory hazards.

HEAs are known for their distinctive properties and versatile applications across industries. These alloys, composed of multiple principal elements in nearly equiatomic proportions, exhibit exceptional mechanical, thermal, and corrosion-resistant characteristics. However, the synthesis and processing of HEAs involve the use of hazardous materials, prompting concerns about their environmental impact and safety. This review identifies the hazardous materials associated with HEAs, explores their potential risks, and discusses ongoing efforts to address environmental and safety concerns while encouraging continued innovation in this promising field [44]. The different classes of elements commonly used in HEAs are briefly presented in Figure 4 and discussed thereafter.

(i). **Reactive metals:** Reactive metals constitute a category of metallic elements characterized by a pronounced inclination to engage in chemical reactions with other substances, notably oxygen and moisture, resulting in the formation of oxides. This reactivity stems from low electronegativity and a distinct tendency to attain a stable electron configuration. Examples of reactive metals include alkali metals (e.g., sodium and potassium), alkaline earth metals (e.g., calcium and magnesium), as well as transition metals such as titanium and zirconium [53]. Major properties of hazardous reactive metals such as Ti, Zr, Hf, Mg, and Al, which exhibit high affinity for oxygen/nitrogen and can cause fire or explosion hazards in fine powder form in Figure 5.
(ii). **Toxic elements:** Toxic elements refer to chemical elements that, when present in specific amounts or configurations, can harm living organisms, including humans, animals, and plants. These elements can have detrimental effects on both human health and ecosystems by disrupting biological processes and interfering with typical physiological functions. The influence of toxic elements varies based on factors such as concentration, exposure duration, and the chemical state in which they are present. Prior studies have examined toxic elements, exploring their origins and impacts and proposing approaches to mitigate their adverse effects [54]. Major properties of hazardous toxic elements (e.g., Ni, Co, Cr, V) associated with respiratory disorders, organ damage, and carcinogenicity upon occupational exposure are shown in Figure 6.
(iii). **Rare-earth elements:** Rare-earth elements possess hazardous properties, including the presence of radioactive isotopes (such as thorium and uranium), toxicity at high concentrations (notably europium and terbium), the release of toxic or flammable gases due to chemical reactivity, respiratory hazards due to fine-particle generation during mining and processing, and the potential for water contamination through runoff from industrial activities. Effective safety measures, environmentally responsible practices, and proper waste management are crucial for mitigating these hazards and minimizing the adverse effects of these elements on human health and the environment [55,56,57]. Major properties of rare-earth elements (e.g., Ce, Y, La) that may induce lung fibrosis, oxidative stress, and bioaccumulation, posing long-term health hazards in laboratory environments, are shown in Figure 7.

## 3. Hazardous Effects and Related Diseases

**Figure 8:**
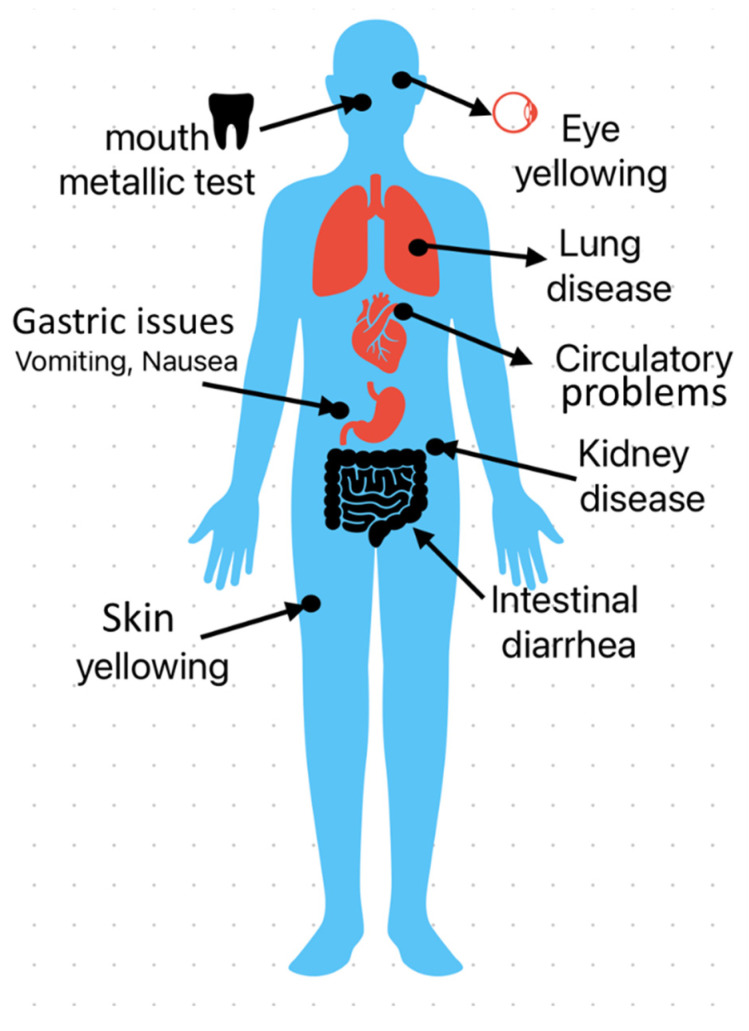
Health hazards are associated with exposure to different metal powders.

Exposure to metal powders can cause respiratory irritation, skin damage, gastrointestinal problems, and long-term health concerns such as organ damage and cancer. The exposure can be in the form of inhalation, touch with the skin, and consumption of metal particles, specifically elements that act as a slow poison to the body, such as aluminum; these elements accumulate inside the body over time, causing major health problems. In addition, while certain heavy metals, such as zinc, copper, chromium, iron, and manganese, are necessary in small amounts for healthy bodily function, excessive concentrations can be hazardous. Some of the major health hazards associated with metal powders are discussed below and presented in Figure 8 [44,58,59,60].

Preventing these health risks necessitates stringent adherence to safety protocols, the use of PPE, adequate ventilation, and compliance with the permissible exposure limits for the relevant metals [61]. Employers and lab personnel should be informed about the potential dangers associated with handling metal powders and should adopt appropriate precautionary measures to safeguard their health [9]. Regular health monitoring and medical supervision are also advisable for those at a higher risk of exposure. A brief description of related diseases and their effect is presented in Table 2.

## 4. Risk Assessment Methodology

### 4.1. Simple Risk Scoring Model

The Simple Risk Scoring Model, also known as the Hazard Ranking Approach, is a qualitative method used to assess potential health risks associated with exposure to hazardous substances. This method assigns numerical values to different risk factors, enabling a comparative ranking of materials based on their toxicity and health hazards. It is widely used in cases where detailed exposure data is unavailable, but a relative assessment of risks is necessary [62,63].

### 4.2. Methodology

The risk assessment in this review is based on two key parameters: toxicity score (TS) and carcinogenicity factor (CF). TS reflects the toxic effects of a metal, considering bio accessibility, organ toxicity, and systemic impacts, based on available toxicological data. A higher TS indicates greater potential harm to laboratory workers exposed to the metal. The risk scoring model in this review evaluates the potential health hazards of metal powders based on two key parameters: TS and CF. TS is assigned by considering the metal’s bioaccessibility, organ-specific toxicity, and systemic impacts, using available toxicological data. Metals with higher toxic effects receive higher TS values on a scale of 1 (low) to 5 (high). CF is determined according to the International Agency for Research on Cancer (IARC) classification, where Group 1 (carcinogenic to humans) = 5, Group 2A (probably carcinogenic) = 4, Group 2B (possibly carcinogenic) = 3, and Not classified = 1. The Risk Score (RS) is calculated as the product of TS and CF (RS = TS × CF), providing a quantitative measure of the overall hazard of each metal. The risk assessment in this review is in Table 3.

The Risk Score (RS) is calculated using the Formula:

RS=TS×CF

where RS represents the overall risk level of the material [65,66]. 

Table 4 lists the metals studied, their TS and CF values, threshold limit values (TLV), and resulting RS. This stepwise approach ensures that the model can be easily reproduced and applied by other researchers for assessing occupational exposure risks in laboratory environments. The following table (Table 4) provides input data for this review. It includes information about metals, their toxicity scores, carcinogenicity classifications, and threshold limit values (TLV) [38,39]. In addition, the purpose of including TLV values was not to contribute to the RS computation, but rather to provide a comparative benchmark between the calculated risk potential (RS) and established occupational exposure limits. This comparison helps highlight cases where metals with high RS also exhibit very low TLVs (e.g., Cr, Cd, Ni), underlining their practical significance in occupational health.

### 4.3. Discussion on Risk Assessment of Metallic Elements

**Figure 9:**
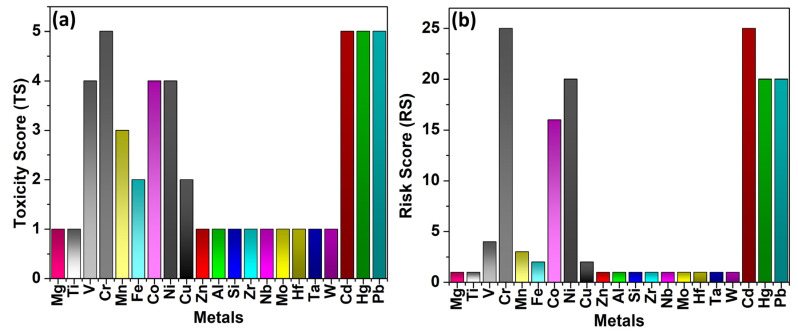
(**a**) Toxicity and (**b**) risk score of different metal powders.

The risk assessment results, when visualized in a bar graph (shown in Figure 9), highlight the varying toxicity levels of different metallic elements (Figure 9a). High-risk metals such as Chromium (Cr), Cadmium (Cd), Lead (Pb), Nickel (Ni), and Mercury (Hg) exhibit the highest Risk Scores (RS ≥ 20) due to their strong toxicity and carcinogenic properties (IARC Group 1 and Group 2A). These metals are associated with severe health effects, including lung cancer, neurological disorders, and kidney damage, particularly in occupational settings like powder metallurgy and nanomanufacturing. Their high RS values emphasize the need for strict exposure controls, proper ventilation, and protective equipment to minimize potential health risks, which is shown in Figure 9b.

Elevated to moderate-risk metals such as Cobalt (Co) and Vanadium (V) (RS of 16 and 4, respectively) still pose significant health concerns, with Cobalt metal classified as a probable human carcinogen (Group 2A). Prolonged exposure can lead to respiratory and cardiovascular complications, necessitating careful handling and regulatory oversight. In contrast, low-risk elements (RS ≤ 3), including Manganese (Mn), Iron (Fe), Copper (Cu), Aluminum (Al), and Titanium (Ti), exhibit minimal toxicity concerns but may still pose health hazards at high exposure levels. A graphical representation of RS values clearly differentiates between high, moderate, and low-risk metals, reinforcing the need for tailored safety measures based on toxicity severity.

These findings underscore the necessity of regulatory intervention, workplace safety policies, and further research on long-term exposure effects, particularly for metal powders and nanoparticles. By integrating risk assessment into occupational health protocols, industries can mitigate hazards, ensuring safer handling of materials in powder metallurgy, biomedical applications, and nanomanufacturing. The presented methodology provides a structured approach to toxicity evaluation, aiding in risk communication and regulatory decision-making.

## 5. Essential Precautions and Prevention Measures

Working with metal powders involves several safety and health risks due to the fine particle size of the powders, the potential for dust generation, and the specific properties of the metals involved. To ensure safety, lab personnel must take appropriate precautions when handling, processing, or working with metal powders.

First and foremost, engineering controls such as local exhaust ventilation (LEV) systems, fume hoods, and enclosed workspaces should be used to minimize airborne dust exposure. The use of glove boxes or sealed containers can further prevent the dispersion of fine particles. Proper personal protective equipment (PPE), including respirators (N95 or higher-rated masks), gloves, lab coats, and safety goggles, must be worn at all times to prevent inhalation, skin contact, or accidental ingestion. Powder handling should be conducted in a designated area with controlled access, and tools such as anti-static brushes or scoops should be used to minimize particle disturbance. Additionally, using wet methods or HEPA-filtered vacuum cleaners instead of compressed air for cleaning prevents powder dispersion and reduces inhalation risks.

In addition to physical safety measures, strict procedural controls must be followed. Scholars should undergo proper training on safe handling, emergency response, and waste disposal methods. Material safety data sheets (MSDS) should be reviewed before working with any new metal powder to understand its hazards, reactivity, and recommended safety precautions. Regular air quality monitoring should be performed to assess exposure levels, and any spills should be contained and cleaned up using appropriate methods. Fire hazards must also be considered, particularly for reactive or combustible powders like aluminum, magnesium, or titanium, which require proper storage in airtight containers and handling away from ignition sources. By adhering to these precautions, lab personnel can work safely with metal powders while minimizing health and environmental risks. Some key safety measures and risk-prevention strategies are discussed in the following section.

### 5.1. Prevention Measures

To ensure the safety and well-being of individuals working with hard metals, cobalt levels in the atmosphere must be maintained below the TLV of 0.3 mg/m^3^. Additionally, individuals with a history of allergic complaints or respiratory diseases should ideally not be employed for manufacturing hard metals. Job applicants should undergo a chest X-ray before commencing work, and X-rays should be performed every two years for all exposed individuals. Similar medical precautions should be exercised for tasks involving the continuous grinding of tungsten carbide. Although routine lung-function tests have been suggested in some countries, they are yet to be widely accepted. Regarding lead, preventing absorption or poisoning necessitates adherence to the TLV of 0.15 mg/m^3^, along with the implementation of necessary precautionary measures, such as Personal protective equipment (PPE) and general hygiene practices [57]. Regular assessments of lead levels in the blood are recommended as a desirable medical precaution.

For other metals, the TLVs are as follows: nickel, 1 mg/m^3^; chromium, 1 mg/m^3^; and cadmium, 0.2 mg/m^3^. For scenarios involving continued exposure to cadmium, routine urine testing should be conducted to detect excess protein. Overall, these measures aim to prevent occupational hazards and ensure the safety of individuals working with these metals [67].

To mitigate danger, strict control and safety precautions are necessary in laboratories where metal powders are handled. To reduce exposure to airborne particles, measures such as fume hoods or local exhaust ventilation systems must be implemented. The workers should also wear the appropriate PPE (Table 5), such as safety goggles, gloves, and respirators. Employees should be trained thoroughly in safe handling techniques and hazard awareness through comprehensive training programs. Maintaining a clean workplace and preventing the buildup of metal dust requires strict adherence to housekeeping procedures and decontamination guidelines. To detect potential risks and ensure that the control measures are functional, risk assessments along with monitoring of air quality and exposure levels are crucial. Moreover, emergency response protocols must be established to handle accidents. Table 6 lists some important control measures for lab environments.

Appropriate fire-extinguishing equipment and fire-suppression systems must be provided, especially for highly reactive metals. In addition, PM laboratories often involve high-energy processes such as ball milling, compaction, and sintering, which generate significant noise and vibration. Noise and vibration are significant occupational hazards in laboratories handling metallic powders. Noise exposure can lead to loss of hearing power and induce stress. Studies also indicate that combined exposure to noise and vibration increases fatigue and accelerates hearing damage, emphasizing the need for proper engineering controls, protective equipment, and regular health monitoring [69,70]. Prolonged exposure to elevated noise levels from mechanical alloying equipment (e.g., planetary ball mills and attritors) can pose occupational hazards, including hearing loss and increased stress levels. Additionally, vibratory compactors and ultrasonic sieving devices contribute to hand-arm and whole-body vibration exposure, potentially leading to musculoskeletal disorders over time. Quantifying noise levels (in decibels, dB) and vibration exposure (measured in acceleration, m/s^2^) is essential for risk assessment and the implementation of protective measures, such as soundproof enclosures, vibration isolation mounts, and personal protective equipment (PPE). Integrating real-time monitoring systems in PM laboratories can help mitigate these occupational hazards and improve workplace safety. A guideline based on the OSHA standard for noise and vibration has been given in Table 7 for different processes.

### 5.2. Health Monitoring

Regular health monitoring and medical surveillance must be conducted for lab personnel handling hazardous metal powders. Access to medical facilities should be provided for immediate treatment in case of exposure or accidents [74,75].

(i). **MSDS:** MSDS or safety data sheets must be maintained for all metal powders used in the workplace, and they should be readily available to research scholars.
(ii). **Regulatory compliance:** Individuals must comply with department rules, university authorities, and standards related to the handling and disposal of hazardous materials.

**Figure 10:**
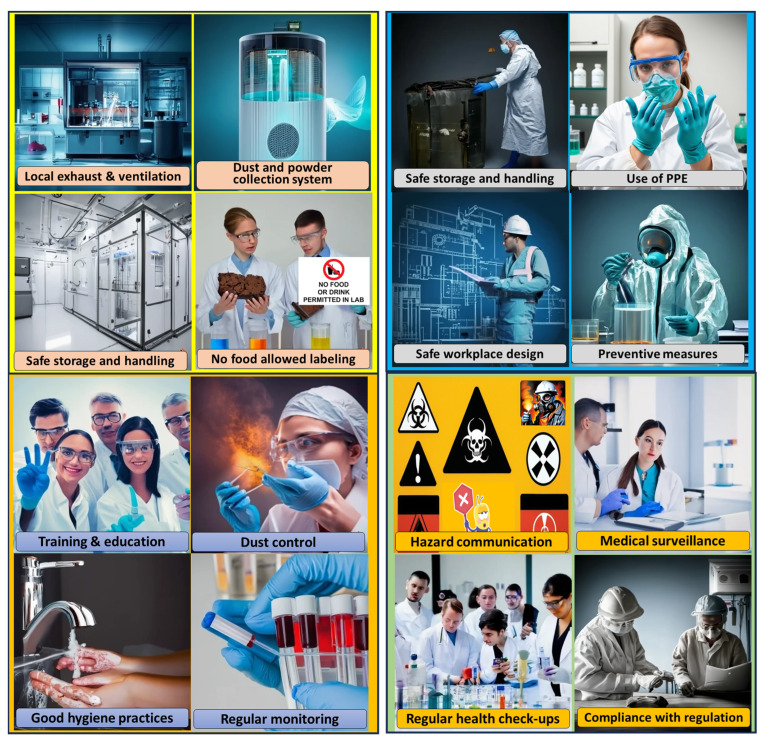
Safety measures to be followed while synthesizing HEAs and working in the powder metallurgy lab.

Lab personnel must always consult with the organization’s safety officer, the safety guidelines, and the relevant regulations to ensure they follow the most up-to-date and appropriate safety practices when working with metal powders. Additionally, they must seek guidance from experts or safety professionals if they are unsure about specific precautions for a particular metal or process. Safety should be the top priority when working with potentially hazardous materials, such as metal powders. In addition, toxins can enter the digestive tract through ingestion if a lab researcher eats, drinks, smokes, or handles contaminated food or food that has been stored in contaminated conditions. Splashes, spills, and airborne particles can cause eye contact and cause irritation or more serious damage to the eyes. It is essential to comprehend these points of entry to put in place practical safeguards to protect the health of lab researchers, as given in Figure 3. Thus, handling different chemicals and fine metallic powders, which can enter the body and have multiple negative health effects, is part of working in a powder metallurgy lab. An outline of the possible effects of these chemicals and how they can enter the body is provided in detail in the figure. By being aware of these potential entry points and their associated health risks, researchers in powder metallurgy labs can reduce their exposure to chemicals and the resulting health effects. An ideal environment and related safety tasks have been presented in Figure 10. In addition, Regular health monitoring is essential for laboratory personnel handling hazardous metal powders to detect early signs of exposure and mitigate potential health risks. The following table (Table 8) summarizes key aspects of medical surveillance, testing indicators, and recommended testing frequency.

## 6. Knowledge Gaps and Future Perspectives

The present review underscores the importance of certain specific avenues for future research on the hazardous effects of working with metal powders, particularly for newly employed lab personnel. Numerous studies have investigated lab environments and industrial areas where powders are produced and handled, and the findings highlight noticeable hazards and health risks. This implies the need for comprehensive studies on the working environments in laboratories and metallurgical processes.

**Figure 11:**
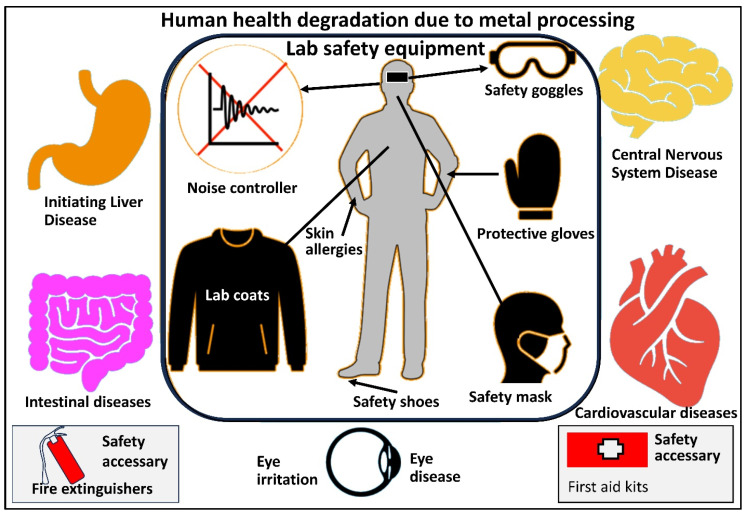
Laboratory safety equipment used in a powder metallurgy lab and the potential health risks associated with exposure to metal powders and other hazardous materials during experimentation.

Ensuring a correlation between the actual safety of a working environment and the health hazards detected in that environment is essential for validating the safe practices identified through critical analysis. Given the wide array of safe work environments available, it becomes necessary for new lab personnel to understand the appropriate way of working in a lab environment and handling different chemical and metal powders to cultivate confidence and produce better research results. A schematic presentation of lab safety equipment and disease is shown in Figure 11.

## 7. Conclusions

In conclusion, metals such as nickel, copper, tungsten, chromium, and cobalt pose significant health risks due to their high bioaccessibility in the human gastric and intestinal phases, making careful handling essential in laboratory experiments. A systematic approach to investigating these hazards is crucial for ensuring safe research practices, with continuous health monitoring of lab personnel being necessary to prevent illnesses. Despite the importance of safety, there is a lack of studies examining the correlation between safety protocols and health risks, highlighting the need for standard procedures when handling metal powders. This review underscores the health risks of HEA processing and introduces a simple risk-scoring model to assess hazards based on toxicity, exposure limits, and carcinogenicity. The model serves as a valuable tool for identifying and mitigating high-risk exposures, thereby improving safety practices. Future research should refine the model by integrating experimental toxicity data and real-world exposure scenarios to enhance its applicability in laboratory environments. Although this review emphasizes exposure risks within powder metallurgy research laboratories, it is important to note that similar hazards may also extend to industrial and manufacturing environments where HEA powders are synthesized, processed, or machined, thereby posing potential risks to both laboratory personnel and factory workers.

Future research should focus on improving existing risk assessment models by integrating quantitative exposure data, advanced toxicological indicators, and predictive tools such as machine learning. Real-time monitoring of airborne particles and biological markers could provide dynamic risk evaluations, while long-term epidemiological studies would help validate model predictions. Additionally, developing standardized databases for toxicity and carcinogenicity will further strengthen the reproducibility and applicability of risk assessment frameworks in laboratory settings.

## Figures and Tables

**Table 1:** Permissible and recommended exposure limits (PEL and REL) by OSHA and NIOSH for different common metals [38,39].

Metal	OSHA PEL	NIOSH REL	Primary Health Effects
**Aluminum (Al)**	15 mg/m^3^ (total dust); 5 mg/m^3^ (respirable fraction)	10 mg/m^3^ (total dust); 5 mg/m^3^ (respirable fraction)	Respiratory issues, lung fibrosis
**Iron (Fe)**	10 mg/m^3^ (as iron oxide fume)	5 mg/m^3^ (as iron oxide fume)	Respiratory irritation, benign pneumoconiosis
**Manganese (Mn)**	5 mg/m^3^ (ceiling limit)	1 mg/m^3^ (TWA); 3 mg/m^3^ (STEL)	Neurological effects, manganism
**Zinc (Zn)**	5 mg/m^3^ (fume); 15 mg/m^3^ (total dust); 5 mg/m^3^ (respirable fraction)	5 mg/m^3^ (TWA); 15 mg/m^3^ (ceiling, 15 min)	Metal fume fever, respiratory irritation
**Tungsten (W)**	5 mg/m^3^ (insoluble compounds)	5 mg/m^3^ (TWA); 10 mg/m^3^ (STEL)	Respiratory irritation
**Silicon (Si)**	15 mg/m^3^ (total dust); 5 mg/m^3^ (respirable fraction)	Not established	Respiratory issues with prolonged exposure
**Copper (Cu)**	1 mg/m^3^ (dusts and mists); 0.1 mg/m^3^ (fume)	1 mg/m^3^ (dusts and mists); 0.1 mg/m^3^ (fume)	Respiratory irritation, gastrointestinal distress
**Titanium (Ti)**	Not established	Not established	Generally considered low toxicity, titanium dioxide dust may cause respiratory irritation.
**Hafnium (Hf)**	0.5 mg/m^3^	0.5 mg/m^3^	Eye and skin irritation, respiratory issues
**Niobium (Nb)**	Not established	Not established	Limited data; potential respiratory effects
**Tantalum (Ta)**	5 mg/m^3^	5 mg/m^3^	Lung effects, fibrosis with prolonged exposure

**Table 2:** List of Diseases Caused by Exposure to Hazardous Substances or Conditions and Their Potential Underlying Causes [44,58,59,60].

Disease	Description
**(i) Respiratory concerns**	The utilization of metal powders in various industries can lead to health issues among lab personnel. Inhaling metal powders, especially fine particles, may result in respiratory complications, such as coughing, shortness of breath, and lung diseases, including fibrosis. Certain metals, such as aluminum and cobalt, have been linked to severe lung diseases.
**(ii) Systemic and organ-specific toxicity risks**	Metal powders, particularly those containing lead and cadmium, can have toxic effects when they enter the body. Lead, in particular, can cause lead poisoning, which impacts the nervous system and various other organs.
**(iii) Allergic reactions**	Working with certain metal powders can trigger allergic responses in susceptible individuals. For instance, cobalt exposure has been associated with acute asthma in sensitive individuals.
**(iv) Skin sensitization**	Contact with metal powders can result in skin irritation, rashes, and dermatitis, especially under prolonged or recurrent exposure.
**(v) Cancer susceptibility**	Prolonged exposure to specific metal powders, such as nickel and chromium, is linked to an increased risk of cancer, particularly in industrial environments.
**(vi) Cardiovascular implications**	Fine particulate matter, including metal powders, can enter the bloodstream and cause cardiovascular problems.
**(vii) Digestive disturbances**	Inadvertently ingesting metal powders, often through hand-to-mouth contact, can lead to gastrointestinal issues.
**(viii) Radiographic abnormalities**	Certain metals, when deposited in the lungs, may not cause immediate harm but may create distinct patterns on chest radiographs.

**Table 3:** Key parameters for risk assessment [62,63].

	Key Parameters	Description
1	Toxicity Score (TS)	Represents the inherent toxicity of a metal, assigned based on known toxicological effects. A higher score indicates greater toxicity.
2	Carcinogenicity Factor (CF)	Based on the International Agency for Research on Cancer (IARC) classification, carcinogenicity is categorized as follows [64]. Group 1 (Carcinogenic to humans) = 5 Group 2A (Probably carcinogenic) = 4 Group 2B (Possibly carcinogenic) = 3 Not classified = 1

**Table 4:** Toxicity scores, carcinogenicity, TLV, and risk scores for different elements [65,66].

Metal	Toxicity Score	Carcinogenicity	Risk Score	TLV (mg/m^3^)	Explanation
Mg	1	Not classified	1	10.0	**Metal**: Name of the metal being studied. **Toxicity Score**: A qualitative measure of toxicity on a scale of 1 to 5 (1 = low, 5 = high). **Carcinogenicity**: Classification of the metal’s potential as a carcinogen (e.g., Group 1 = carcinogenic to humans, Group 2A = probably carcinogenic, etc.). **TLV (mg/m^3^)**: Threshold Limit Value, indicating the maximum safe airborne concentration in milligrams per cubic meter.
Ti	1	Not classified	1	10.0	
V	4	Not classified	4	0.05	
Cr	5	Group 1	25	0.01	
Mn	3	Not classified	3	0.2	
Fe	2	Not classified	2	5.0	
Co	4	Group 2A	16	0.02	
Ni	4	Group 1	20	0.015	
Cu	2	Not classified	2	1.0	
Zn	1	Not classified	1	5.0	
Al	1	Not classified	1	10.0	
Si	1	Not classified	1	5.0	
Zr	1	Not classified	1	5.0	
Nb	1	Not classified	1	5.0	
Mo	1	Not classified	1	10.0	
Hf	1	Not classified	1	0.5	
Ta	1	Not classified	1	5.0	
W	1	Not classified	1	5.0	
Cd	5	Group 1	25	0.002	
Hg	5	Group 2A	20	0.01	
Pb	5	Group 2A	20	0.05	

**Table 5:** List of personal protective equipment [52,68].

Personal Protective Equipment	Purpose
Safety glasses or goggles	Protect the eyes from splashes, dust, and particles.
Lab coats or protective Clothing	Prevent skin contact with powders.
Respiratory protection	Depending on the particle size and toxicity of the metal powders, use appropriate respirators (e.g., N95 or higher) to prevent inhalation of airborne particles.
Gloves	Use gloves resistant to the specific metal being handled.
Disposable coveralls or dust suits	These can provide additional protection when working with highly hazardous or toxic powders.

**Table 6:** Control measures for the lab environment.

Control Measures	Safe Practices for Handling Metal Powders
Local exhaust ventilation	Install fume hoods or local exhaust systems to capture and remove airborne dust or fumes at the source.
Isolation and containment	Keep metal-powder handling areas separated from other workspaces to prevent contamination.
Dust-collection systems	Use dust collectors or industrial vacuum systems to capture and contain loose powders.
Safe handling practices	Handle all the equipment with patience and mindfulness.
Minimize dust generation	Use techniques such as wetting the powders or working in glove boxes with controlled atmospheres to reduce dust generation.
Avoid spills	Handle powders with care to prevent spills or releases.
No smoking or eating	Prohibit smoking, eating, or drinking in areas where metal powders are handled.
Proper labeling	Clearly label containers with the type of metal powder and any associated hazards.
Regular cleaning	Maintain a clean work environment and regularly clean surfaces to prevent dust buildup.
Training and Education	Ensure that lab personnel are trained in the safe handling of metal powders, including the proper use of PPE and the recognition of potential hazards.
Emergency response	Establish emergency response procedures, including response to spillage and evacuation plans.

**Table 7:** Control measures for noise in the lab environment as per OSHA [71].

Process/Equipment	Noise Level (dB)	Vibration Frequency (Hz)	Limited by OSHA	Remark
**Ball Milling**	80–90	5–15	<85 dB	Noise levels can vary depending on the mill design and operational parameters. Vibration frequencies are typically low, contributing to the overall noise environment.
**Sintering**	60–70	Minimal		Sintering furnaces generally produce low noise and minimal vibration, primarily from auxiliary equipment like fans or conveyors.
**Ultrasonication**	70–85	20,000–40,000		Ultrasonic processors operate at high frequencies (20 kHz and above), with noise levels depending on power settings and enclosure effectiveness [72].
**Cutting/Polishing**	85–95	50–60		Mechanical cutting and polishing tools generate significant noise and vibration, varying with material hardness and equipment type.
**Wire EDM**	75–85	Variable		Wire Electrical Discharge Machining produces moderate noise; vibration levels depend on machine settings and workpiece material [73].

**Table 8:** Key aspects of medical surveillance, testing indicators, and recommended testing frequency.

Aspect	Recommendation	Details/Indicators	Testing Frequency	Ref.
Initial Medical Examination	Baseline assessment	Comprehensive medical and occupational history	Once, before exposure	[39,71]
Complete Blood Count (CBC)	Monitoring hematological changes	Detect systemic toxicity	High risk: Quarterly Moderate risk: Semi-annual Low risk: Annual	[71,76]
Liver Function Tests (LFTs)	Monitor hepatic function	Early detection of liver damage	High risk: Quarterly Moderate risk: Semi-annual Low risk: Annual	[71,76]
Kidney Function Tests	Monitor renal function	Early detection of nephrotoxicity	High risk: Quarterly Moderate risk: Semi-annual Low risk: Annual	[71,76]
Blood Metal Levels	Measure specific metals (e.g., Pb, Cd, Hg)	Assess internal exposure	High risk: Quarterly Moderate risk: Semi-annual Low risk: Annual	[71,77]
Urinary Metal Excretion	Measure metal content in urine	Provides body burden information	High risk: Quarterly Moderate risk: Semi-annual Low risk: Annual	[71,78,79]

## Data Availability

No new data were created or analyzed in this study. Data sharing is not applicable to this article.
